# Activation of CHK1 in Supporting Cells Indirectly Promotes Hair Cell Survival

**DOI:** 10.3389/fncel.2017.00137

**Published:** 2017-05-18

**Authors:** Azadeh Jadali, Yu-Lan M. Ying, Kelvin Y. Kwan

**Affiliations:** ^1^Department of Cell Biology and Neuroscience, Rutgers UniversityPiscataway, NJ, USA; ^2^Stem Cell Research Center and Keck Center for Collaborative Neuroscience, Rutgers UniversityPiscataway, NJ, USA; ^3^3D BiotekBridgewater, NJ, USA; ^4^Department of Otolaryngology—Head and Neck Surgery, Rutgers New Jersey Medical SchoolNewark, NJ, USA

**Keywords:** cisplatin, ototoxicity, cochlea, supporting cell, cell survival, PI3 kinase signaling, AKT, CHK1

## Abstract

The sensory hair cells of the inner ear are exquisitely sensitive to ototoxic insults. Loss of hair cells after exposure to ototoxic agents causes hearing loss. Chemotherapeutic agents such as cisplatin causes hair cell loss. Cisplatin forms DNA mono-adducts as well as intra- and inter-strand DNA crosslinks. DNA cisplatin adducts are repaired through the DNA damage response. The decision between cell survival and cell death following DNA damage rests on factors that are involved in determining damage tolerance, cell survival and apoptosis. Cisplatin damage on hair cells has been the main focus of many ototoxic studies, yet the effect of cisplatin on supporting cells has been largely ignored. In this study, the effects of DNA damage response in cochlear supporting cells were interrogated. Supporting cells play a major role in the development, maintenance and oto-protection of hair cells. Loss of supporting cells may indirectly affect hair cell survival or maintenance. Activation of the Phosphoinositide 3-Kinase (PI3K) signaling was previously shown to promote hair cell survival. To test whether activating PI3K signaling promotes supporting cell survival after cisplatin damage, cochlear explants from the neural subset (NS) Cre *Pten* conditional knockout mice were employed. Deletion of Phosphatase and Tensin Homolog (PTEN) activates PI3K signaling in multiple cell types within the cochlea. Supporting cells lacking PTEN showed increased cell survival after cisplatin damage. Supporting cells lacking PTEN also showed increased phosphorylation of Checkpoint Kinase 1 (CHK1) levels after cisplatin damage. Nearest neighbor analysis showed increased numbers of supporting cells with activated PI3K signaling in close proximity to surviving hair cells in cisplatin damaged cochleae. We propose that increased PI3K signaling promotes supporting cell survival through phosphorylation of CHK1 and increased survival of supporting cells indirectly increases hair cell survival after cisplatin damage.

## Introduction

Sensorineural hearing loss caused by the exposure to loud sounds and ototoxic drugs results in the loss of sensory hair cells and spiral ganglion neurons of the inner ear. Cisplatin is a potent antitumor agent used for its wide clinical activity against many different types of tumors (Wang and Lippard, 2005). A side effect of cisplatin-induced hearing loss is the loss of cochlear hair cells (Rybak et al., 2009; Schacht et al., 2012). Systemic injection of cisplatin results in discernable cisplatin-DNA adducts in hair cells and supporting cells that reside in the organ of Corti (van Ruijven et al., 2005). Supporting cells have distinct morphology and specific anatomical locations within the organ of Corti. Dieters’ cells reside below the outer hair cells (OHC), inner and outer pillar cells form the tunnel of Corti while Hensen’s and Claudius cells are located in the outer sulcus. These cells contribute to the microarchitecture observed in the sensory epithelium (Monzack and Cunningham, 2013; Wan et al., 2013). In addition to maintaining the cytoarchitecture in the cochlea, supporting cells such as inner border cells and inner phalangeal play critical roles in development, maintenance and synaptogenesis of hair cells (Gómez-Casati et al., 2010; Mellado Lagarde et al., 2014). Supporting cells can phagocytose dying hair cells to clear up cellular debris in the sensory epithelium (Bird et al., 2010; Monzack et al., 2015). Supporting cells can protect hair cells from damage by secreting the Heat Shock Protein 70 (HSP70; May et al., 2013). Systemic application of cisplatin shows that supporting cells are structurally damaged and could contribute to delayed hair cell loss (Ramírez-Camacho et al., 2004). Furthermore, after cisplatin damage, phagocytosis of dead hair cells by supporting cells is impaired (Monzack et al., 2015). These studies suggest that supporting cells play distinct cellular roles to maintain proper cochlear function and may be targets of cisplatin damage.

Cytotoxicity after cisplatin exposure is caused by the formation of intra- and inter-strand DNA crosslinks as well as cisplatin DNA adducts (Wang and Lippard, 2005). DNA damage activates several signal transduction pathways that include Ataxia Telangiectasia and Rad3 Related Protein (ATR), p53, p73 and Mitogen-Activated Protein Kinase (MAPK) and results in either cell survival or apoptosis (Siddik, 2003). Checkpoint Kinase 1 (CHK1) is a key downstream target that is phosphorylated by ATR at serine residues 345 and 317 (Zhao and Piwnica-Worms, 2001). Phosphorylation of CHK1 increases kinase activity and initiates the DNA damage response by activating DNA damage repair, DNA damage checkpoints and cell death proteins (Zhang and Hunter, 2014). DNA damage-mediated apoptotic signals can be attenuated as observed in cisplatin resistant tumor cells. Cisplatin resistant cancer cells arise when propagation of the DNA damage signal to the apoptotic machinery is inhibited. These mechanisms include activation of the Phosphoinositide 3-Kinase (PI3K)/RAC-Alpha Serine Threonine Protein Kinase (PI3K/AKT) signaling, loss of p53 function and overexpression of anti-apoptotic factors such as B-cell leukemia/lymphoma 2 (BCL2) to promote cell survival (Siddik, 2003). In this study, we determined whether phosphorylation of CHK1 promotes cochlear supporting cell survival after cisplatin damage.

## Materials and Methods

### Cell Culture

Immortalized multipotent otic progenitor (iMOP) cells were grown in suspension with DMEM/F12 (Life Technologies) containing B27 supplement (Life Technologies), 25 μg/ml carbenicillin and 20 ng/ml basic fibroblast growth factor (bFGF; Pepro Tech; Jadali et al., 2016). For differentiation experiments, cells were cultured for 3 or 7 days in the absence of bFGF depending on the experiment. iMOP cells were treated with LY294002 (LC laboratories), bpV(Hopic; Santa Cruz Biotechnology, Inc., Santa Cruz, CA, USA) or cisplatin (Sigma) at the specified concentrations. LY294002 and bpV(Hopic) were solubilized in dimethyl sulfoxide (DMSO) and added to medium as described. Cisplatin was dissolved into medium and warmed to 37°C to make a 1 mM stock solution. Cisplatin solution was diluted into culture medium at the described concentrations.

### Proliferation and EdU Incorporation Assays

For 5-ethynyl-2′-deoxyuridine (EdU) labeling, the Click-iT EdU Alexa Fluor 488 assay kit (Life Technologies) was used. iMOP cells were pulsed with 1 μM EdU for 2 h. After EdU incorporation, cells were removed from culture, dissociated to generate single cells, fixed, labeled EdU with Alexafluor 488 by click chemistry, resuspended in 1× PBS containing 0.1% Tween 20 and mounted on a slide. Fluorescent images of labeled cells were taken using epifluorescence microscopy and the percentage of EdU positive cells in 1000 nuclei was determined.

### Flow Cytometry and Apoptosis Assay

iMOP cells were differentiated for 3 days before they were treated with 50 μM cisplatin alone, or pre-treated with 25 μM LY294002 or 10 μM bpV(HOpic) for 1 h before treatment with 50 μM cisplatin. Apoptotic cells were analyzed 24 h after treatment with small molecules. To identify apoptotic cells, the Alexa Fluor 488 annexin V/PI dead cell apoptosis kit was used (Life Technologies) according to manufacturer’s instructions. Cells labeled with Alexa Fluor 488 annexin V and/or PI and were quantified by flow cytometry using a Beckman Coulter Gallios flow cytometer with the appropriate filters.

### Western Blot Analysis

Cells were lysed in lysis buffer (50 mM Tris/HCl, pH 7.5, 150 mM NaCl, 1 mM EDTA, 1 mM EGTA, 1% Triton X-100, and 10% glycerol containing phosphatase inhibitor (Thermo Scientific) and a mixture of protease inhibitors (Roche). Protein lysates (30 μg) were loaded and separated on 4%–12% Bis Tris Novax NuPAGE gradient gels (Life Technologies), transferred to PVDF membrane, and incubated in blocking buffer (phosphate-buffered saline (PBS), 0.1% Tween 20 and 5% nonfat dried milk) for 1 h. To detect proteins of interest, membranes were incubated overnight at 4°C with primary antibodies. Immunoreactive bands were detected by incubating with horseradish peroxidase-conjugated secondary antibodies, followed by application of chemiluminescence substrate (Pierce ECL, Thermo Fisher Scientific). Membranes were exposed to either X-ray film (RPI) or Amersham Hyperfilm ECL (GE Healthcare) for signal detection before film development. To detect multiple proteins using the same membrane, membranes were stripped and re-probed with the appropriate primary antibodies. Quantification of the intensity from individual bands was done using Photoshop. Antibodies and dilutions of antibodies for Western blot are described in Table 1.

**Table 1:** **Antibodies and Small Molecules**.

Antibody/Small molecule	Company	Use	Dilution
AKT	Cell Signaling Technologies	Western blot	1:3000/1:1000
pAKT-Thr308	Cell Signaling Technologies	Western blot	1:1000
CHK1	Cell Signaling Technologies	Western blot	1:1000
pCHK1-Ser345	Cell Signaling Technologies	Western blot/Immunostaining	1:1000/1:100
ACTB	Santa Cruz Biotechnology	Western blot	1:1000
MYO7A	Proteus	Immunostaining	1:1000
Phalloidin-Alexafluor 647	Life Technologies	Immunostaining	1:400

### Cochlear Explant Cultures

Cochleae from P4–6 pups were dissected and cleaned of surrounding tissue and bone. The stria vascularis was trimmed and the Reissner’s and the tectorial membrane peeled away to expose the sensory epithelium. To ensure that the explanted cochlea adhered to the bottom of the coverglass, the cochlea was cut into three individual pieces and adhered onto a 1.5 cover glass treated with 10 μg/ml poly-L-ornithine. Cochlear explants were cultured in DMEM/F12 containing 10% FBS, 2 mM L-Glutamine and 25 μg/ml carbenicillin. One day after plating, cochleae were treated with either 10 μM bpV(HOpic) or DMSO before addition of 20 μM cisplatin. Hair cells counts per 200 μm were divided into base, middle and apical regions of the cochlear explants and used to determine the percent of surviving hair cells along the tonotopic axis.

### Immunofluorescence Staining

Conditions used for immunostaining of iMOP cells were previously described (Kwan et al., 2015). Cochlear explants were fixed in 4% formaldehyde with 1× PBS for 1 h, permeabilized in wash buffer (PBS and 0.1% Triton X-100) for 10 min, incubated in blocking buffer (PBS, 10% goat serum and 0.1% Triton X-100) for 1 h and incubated overnight with a 1:500 dilution of Myosin 7A (MYO7A; Proteus Biosciences) primary antibody in blocking buffer. For CHK1 labeling, antigen retrieval was accomplished by incubating fixed cochlea in 150 mM Tris-HCl at pH 9.0 for 5 min, followed by heating at 70°C for 15 min. Cochleae were rinsed in wash buffer before incubating overnight with a 1:100 dilution of pCHK1 (Cell Signaling) antibodies in blocking buffer. Detection of primary antibodies was done by removing the primary antibody solutions and washing the cochleae in wash buffer three times before incubating in 1:500 dilution of goat anti-rabbit Alexa Fluor 488 secondary antibody and 1:500 phalloidin Alexa Fluor 647 (Life Technologies) in blocking buffer for 2 h. Cochleae were rinsed and mounted on slides with prolong gold antifade mounting media (Life Technologies). Immunofluorescence images were obtained using a Zeiss 510 confocal microscope with a 40 × 1.3 NA water immersion objective. The average percent of MYO7A positive inner hair cells (IHC) and OHC were obtained by calculating the percentage of MYO7A cells along the cochlear axis from treated cochlear explants relative to untreated controls. Antibodies and dilutions of antibodies for immunostaining are described in Table 1.

### Biohazardous Material

All biohazardous material such as LY294002, bpV (HOpic) and cisplatin were handled in a laminar flow hood and appropriately disposed after usage as recommended by Rutgers Environmental Health and Safety (REHS).

### Animals

All mice were housed in the Nelson Labs animal facility in microisolator cages. Neural subset (NS) Cre *PTEN* mice in the B6;129S4 genetic background were previously described (Jadali and Kwan, 2016). This study was carried out in accordance with the recommendations of the Rutgers Animal Care and Facilities Committee (ACFC). The protocol was approved by the Rutgers University Institutional Animal Care and Use Committee (IACUC).

### Statistical Analysis

All error bars shown in data are expressed as ± standard deviation (SD) of values obtained from independent experiments unless otherwise stated. The numbers (*n*) of independent experiments or individual wells from separate cultures are listed for experiments. Technical triplicates were included in each experiment. An unpaired two-tailed Student’s *t-test* was used to determine statistical significance and associated with the appropriate *p* value. For all figures *p* values are defines as: *p < 0.05, **p < 1 × 10^−2^, ***p < 1 × 10^−3^ and ****p < 1 × 10^−4^ unless otherwise stated.

## Results

### Differentiating iMOP Cells Express Hair Cell and Supporting Cell Markers

**Figure 1:**
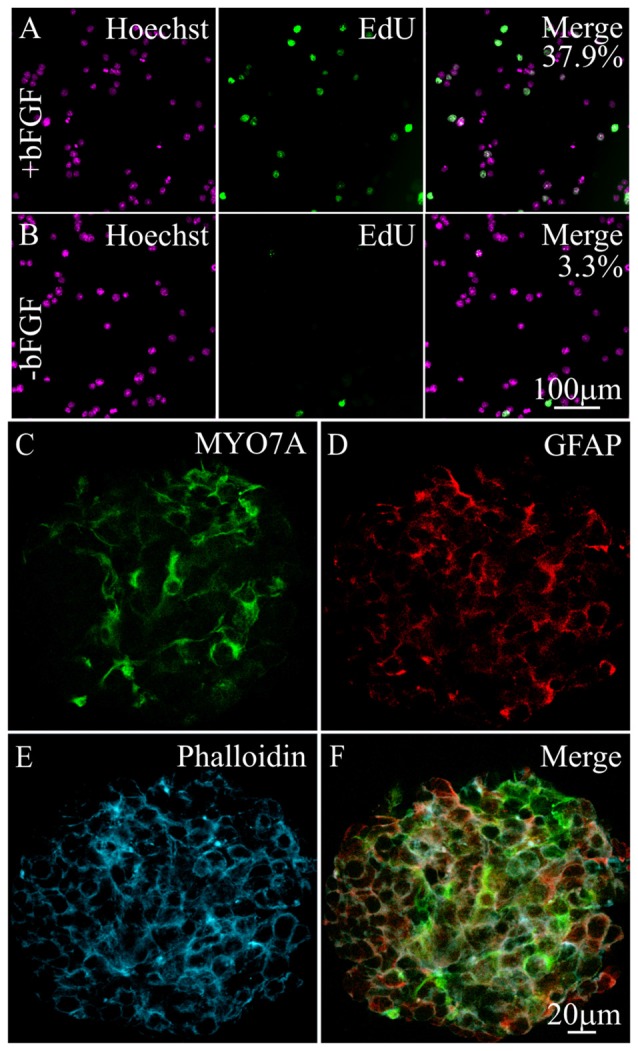
**Expression of hair cell and supporting cell markers in differentiating immortalized multipotent otic progenitor (iMOP) cells**. Proliferating iMOP cells cultured in basic fibroblast growth factor (bFGF) were subjected to 5-ethynyl-2′-deoxyuridine (EdU) incorporation. **(A)** Hoechst labeled nuclei from proliferating iMOP cells show EdU incorporation in 37.9% of cells (*n* = 3). **(B)** Hoechst labeled nuclei from differentiating iMOP cells showed EdU incorporation in 3.3% of cells (*n* = 3). Differentiating iMOP cells express **(C)** MYO7A and **(D)** glial fibrillary acidic protein (GFAP) in **(E)** phalloidin marked otospheres. **(F)** Otospheres from differentiating iMOP cultures were used to test for the effects of cisplatin treatment.

To identify signaling pathways that maintain hair cell survival, we used iMOP cells that can self-renew and differentiate into hair cells and supporting cells (Kwan et al., 2015). Differentiating iMOP cells were generated by withdrawing bFGF for 7 days, to promote cell cycle exit and differentiation (Jadali et al., 2016). To determine the proliferative capacity of proliferating or differentiating iMOP cultures, incorporation of the nucleotide analog EdU was used. As iMOP cells progress through the cell cycle and undergo DNA replication, EdU was incorporated into the DNA. Incorporation of EdU provides an index for proliferation. Proliferating iMOP cells and differentiating iMOP cells normally grow as clusters of cells, or otospheres. To allow for unambiguous cell counts, otospheres from iMOP cultures were dissociated, fixed and labeled with Hoechst after EdU incorporation. Proliferating iMOP cells showed EdU labeling in 37.9% ± 2.5 of Hoechst labeled nuclei (Figure 1A). Differentiating iMOP cells showed EdU labeling in 3.3% ± 1.2 of Hoechst labeled nuclei (Figure 1B). A significant 11.5 fold reduction (*p* < 1 × 10^−4^) in cells that have undergone DNA replication was observed in differentiating cells compared to proliferating cells. These results suggest that the vast majority of differentiating iMOP cells were no longer progressing through the cell cycle.

To determine the extent of differentiation, otospheres from differentiating iMOP cultures were harvested, fixed and immmunostained with antibodies against MYO7A, a hair cell marker and glial fibrillary acidic protein (GFAP), a supporting cell marker. Differentiating iMOP cells showed MYO7A (Figure 1C) and GFAP (Figure 1D) labeling. Otospheres from differentiating iMOP cells also showed circumferential actin after phalloidin labeling (Figure 1E) that was reminiscent of actin filament distribution in the developing cochlea sensory epithelium. Differentiating iMOP cultures containing MYO7A and GFAP expressing cells were subsequently employed as a cellular platform to study the effects of cisplatin damage in hair cells and supporting cells (Figure 1F).

### Activation of PI3K Promotes iMOP Cell Survival after Cisplatin Damage

**Figure 2:**
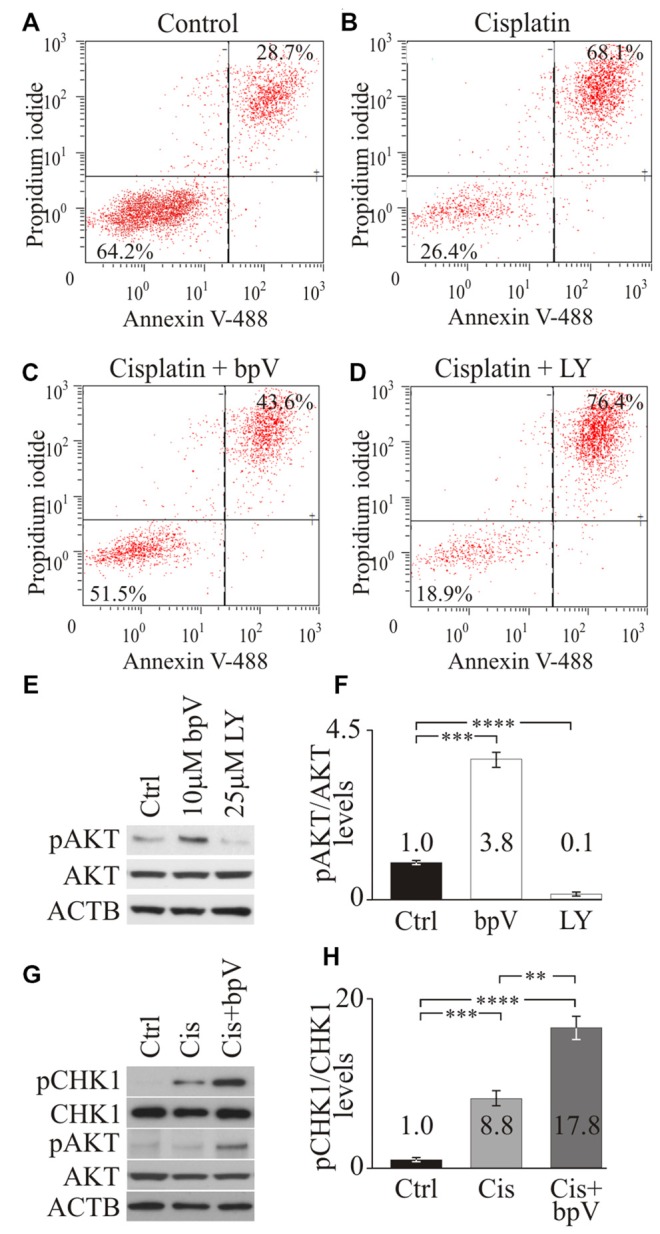
**Checkpoint Kinase 1 (CHK1) phosphorylation after activating phosphoinositide 3-kinase (PI3K) signaling**. Propidium iodide (PI) and annexin V FACS analysis of **(A)** control dimethyl sulfoxide (DMSO), **(B)** cisplatin **(C)** cisplatin and bpV(HOpic) and **(D)** cisplatin and LY treated cells from representative plots. Average percentage of cells from viable (PI- annexin V-) and apoptotic (PI+ annexin V+) quandrants were displayed (*n* = 4). **(E)** Western blot of phospho-AKT (pAKT), total AKT and ACTB in control DMSO, 10 μM bpV(HOpic) or 25 μM LY294002 treated cells (*n* = 4). **(F)** Quantification of pAKT/AKT ratio from Western blots (*n* = 4). **(G)** Western blot of pCHK1, total phospho-CHK1 (CHK1), pAKT, total AKT and ACTB in control DMSO, 20 μM cisplatin, 20 μM cisplatin and 10 μM bpV(HOpic) treated cells (*n* = 4). **(H)** Quantification of pCHK1/CHK1 ratios from Western blots (*n* = 4). Statistical significance was determined using the Student’s *t-test* and error bars are in standard deviation (SD).

Since PI3K signaling has been implicated in maintaining hair cell survival after aminoglycoside damage (Chung et al., 2006; Jadali and Kwan, 2016), we determined whether PI3K signaling was also involved in promoting supporting cell survival after cisplatin damage. To test how PI3K signaling affects cell survival, differentiating iMOP cells were treated with small molecules that activated or inhibited PI3K signaling. To activate PI3K/AKT signaling, we used the small molecule bpV(HOpic) that inhibits phosphatase tension homolog deleted on chromosome 10 (*PTEN*) and increase phosphatidylinositol-3,4,5-trisphosphate (PIP_3_) levels. Excess PIP_3_ at the cell membrane increases PI3K signaling (Cantley and Neel, 1999; Doillon et al., 1999; Schmid et al., 2004). As a control, the small molecule LY294002 was used as an inhibitor of PI3K activity (Vlahos et al., 1994). The concentrations of small molecules employed was previously identified from dose response curves (Jadali and Kwan, 2016). Activator or inhibitor of PI3K signaling was added before cisplatin-induced damage in differentiating iMOP cells and apoptotic cells were quantified by flow cytometry analysis. Differentiating iMOP cells were treated with cisplatin, harvested and subjected to dual labeling with propidium iodide (PI) and Alexafluor 488-conjugated annexin V. Treated samples were compared to DMSO treated controls. FACS analysis was employed to identify apoptotic cells labeled with PI and apoptotic marker annexin V (Vermes et al., 1995). The percentage of viable cells (PI- annexin V) in the lower left quadrant and the apoptotic cells (PI+ annexin V+) in the upper right quadrant of the FACS plot were quantified. In control, 64.2% viable cells and 28.7% apoptotic cells were observed (Figure 2A). Treatment with 50 μM cisplatin in differentiating iMOP cells resulted in 26.4% viable cells and 68.1% apoptotic cells (Figure 2B). These results suggest an increase in apoptotic cells after cisplatin treatment.

To test whether activation of the PI3K pathway would promote cell survival after cisplatin treatment, differentiating iMOP cells were cultured with 10 μM of bpV(HOpic) for 1 day before cisplatin treatment. Pre-treatment with bpV(HOpic) resulted in 51.5% viable cells and 43.6% apoptotic cells (Figure 2C). Compared to cisplatin treatment alone, addition of bpV(HOpic) increased the percentage of viable cells. These results suggested that addition of bpV(HOpic) promotes cell survival even after exposure to cisplatin. To implicate the role of PI3K signaling in cell survival, LY294002, an inhibitor for PI3K activity was used. Differentiating iMOP cells were pretreated with 25 μM LY294002 before cisplatin treatment resulted in 18.9% viable cells and 76.4% apoptotic cells (Figure 2D). Inhibition of PI3K signaling increased the percentage of apoptotic cells. Together, these results suggest that activation of the PI3K signaling pathway may attenuate apoptosis and promote cell survival in differentiating iMOP cells.

### Activation of PI3K Increases CHK1 Phosphorylation after Cisplatin Damage

To ensure that small molecules had the appropriate effect on PI3K signaling, phosphorylated RAC-alpha serine/threonine protein kinase (AKT) levels were employed as a measure of PI3K signaling activity. Western blot for phospho-AKT (pAKT), AKT and β-Actin (ACTB) was performed on cell lysates obtained from untreated control, 10 μM bpV(HOpic) treated and 25 μM LY294002 treated differentiating iMOP cultures (Figure 2E). ACTB served as a loading control. To quantify the amount of pAKT and total AKT, Western blot signals from the appropriate bands were quantified and the ratio of pAKT to AKT levels was determined. Signals were then normalized to untreated control and presented as fold change. After treatment with 10 μM bpV(HOpic), a significant 3.8 ± 0.32 fold increase in normalized pAKT levels was observed compared to control (*p* < 1 × 10^−3^). Treatment of differentiating iMOP cells with 25 μM LY294002 showed a significant 10 ± 0.02 fold decrease in normalized pAKT levels compared to control (*p* < 1 × 10^−4^; Figure 2F). These results suggested that 10 μM bpV(HOpic) and 25 μM LY294002 can be used to activate or inhibit PI3K signaling respectively in differentiating iMOP cells.

Following cisplatin damage, CHK1, an evolutionarily conserved protein kinase that regulates DNA damage repair is phosphorylated and activated by ATR (Zhao and Piwnica-Worms, 2001). ATR phosphorylates CHK1 at serine residues 317 and 345 in response to DNA damage (Zhao and Piwnica-Worms, 2001). Phosphorylation of CHK1 at Ser345 serves to localize CHK1 to the nucleus following DNA damage checkpoint activation (Jiang et al., 2003). To determine the effects of PI3K signaling in DNA damage response in differentiating iMOP cells, phosphorylation of CHK1 at Ser345 was determined by Western blot using phospho-specific antibodies. Differentiating iMOP cultures were treated with cisplatin to determine if phospho-CHK1 (pCHK1) levels increased relative to untreated control. To promote cell survival and determine the effects of activating PI3K signaling on pCHK1 levels, differentiating iMOP cultures were pretreated with 10 μM bpV(HOpic) before inducing cisplatin damage. Lysates from iMOP cultures were collected and used for Western blot analysis. ACTB was used as a loading control (Figure 2G). The ratio of pCHK1 and total CHK1 were used to determine normalized pCHK1 levels and represent the extent of DNA damage response. After treatment with 10 μM cisplatin alone, a significant 8.8 ± 1.0 fold increase in normalized pCHK1 levels was observed compared to untreated control (*p* < 1 × 10^−3^; Figure 2H). This suggests that cisplatin damage of differentiating iMOP cells activated the DNA damage response. To determine if activation of PI3K signaling alters pCHK1 levels, differentiating iMOP cultures were pretreated with bpV(HOpic) before cisplatin. Normalized pAKT levels were used as a measure of PI3K signaling activity. As observed in the Western blot, addition of bpV(HOpic) increased normalized pAKT levels 3.2 ± 0.2 fold relative to untreated control and increased 3.5 ± 0.5 fold relative to cisplatin treated cultures (Figure 2G). These results suggest that addition of bpV(HOpic) activates PI3K signaling. Pretreating iMOP cells with 10 μM bpV(HOpic) before incubating in 10 μM cisplatin resulted in a significant increase in pCHK1 levels (Figure 2G). Quantification of the Western blot showed a 17.8 ± 1.5 fold increase in normalized pCHK1 levels compared to untreated control (*p* < 1 × 10^−4^; Figure 2H). Comparing cisplatin treated sample to bpV(HOpic) and cisplatin treated samples, a significant 2.0 fold increase in normalized pCHK1 levels was observed (*p* < 1 × 10^−2^; Figure 2H). The results suggest that activation of PI3K signaling increases phosphorylation of CHK1 after cisplatin damage and could promote survival of differentiating iMOP cells.

### Activation of PI3K Signaling Using bpV(HOpic) Promotes Hair Cell Survival after Cisplatin Damage

The above results suggested that activation of PI3K signaling could enhance CHK1 phosphorylation and promote a DNA damage response to increase cell survival. We previously showed that activation of PI3K signaling could promote hair cell survival (Jadali and Kwan, 2016). We wanted to extend our findings and determine if activation of the PI3K signaling can promote supporting cell survival in the cochlea when subjected to ototoxic damage. Since hair cell survival may depend on the oto-protective effect and maintenance of supporting cells (May et al., 2013; Mellado Lagarde et al., 2014), we used hair cells counts and hair cell organization as an indirect indicator for the presence of supporting cells after challenging cochlea explants with cisplatin.

**Figure 3:**
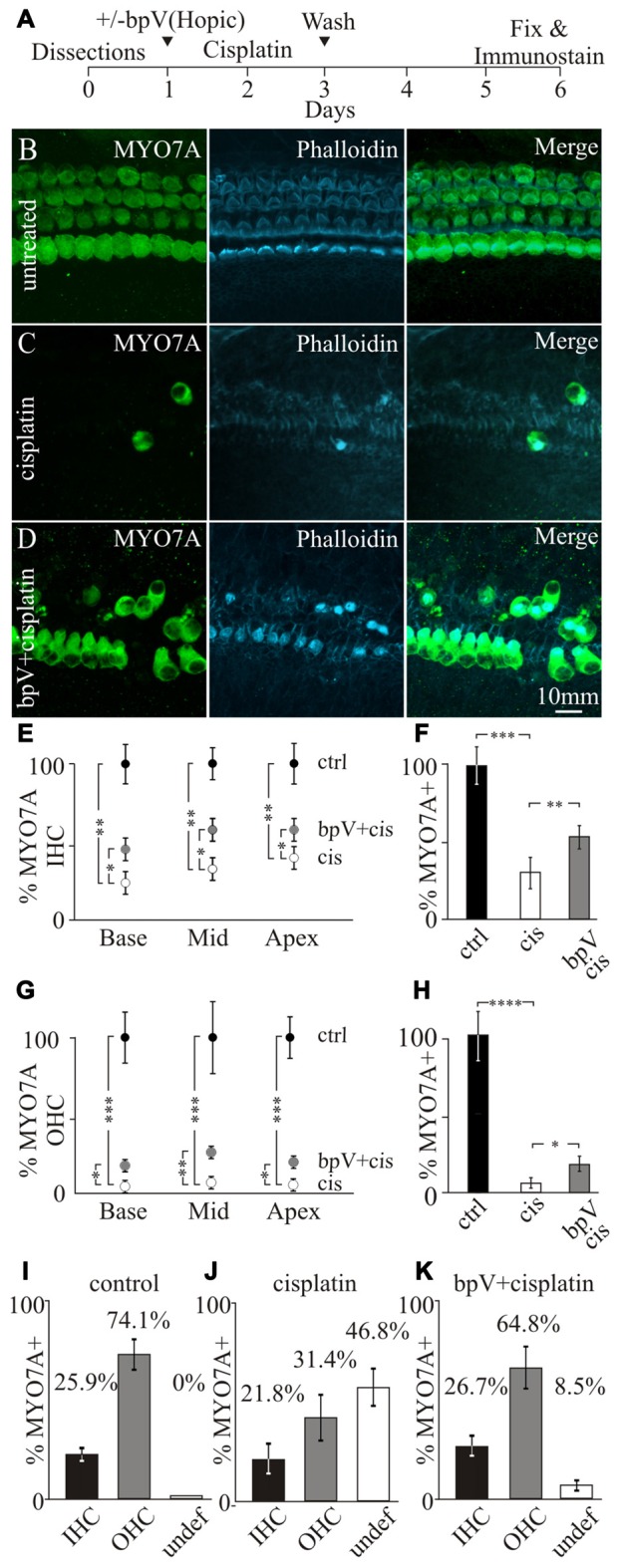
**Effects of bpV(HOpic) on hair cell survival after cisplatin damage. (A)** Timeline of cochlear explant cultures describing the addition of 10 μM bpV(HOpic) 1 day before 10 μM cisplatin treatment and harvesting of explants for immunostaining. Immunofluorescence of MYO7A, phalloidin and merged images from **(B)** control cochlear explant (*n* = 4), **(C)** cochlear explant treated with cisplatin (*n* = 4) and **(D)** cochlear explant pretreated with 10 μM bpV(HOpic) before cisplatin damage (*n* = 4). **(E)** Quantification of MYO7A inner hair cells (IHC) from the basal, middle and apical regions of cochlea explants in control (black), cisplatin (white) and bpV(HOpic)/cisplatin (gray; *n* = 4). **(F)** Average percent of IHC in control (black; *n* = 4), cisplatin (white; *n* = 4) and bpV(HOpic) and cisplatin (gray; *n* = 4) treated explants normalized to control. **(G)** Percentage of MYO7A outer hair cells (OHC) in control (black; *n* = 4), bpV(HOpic) and cisplatin (gray; *n* = 4) and cisplatin treated (white; *n* = 4) cochlear explants from the basal, middle and apical regions. **(H)** Average percent of OHC from control (black; *n* = 4), cisplatin (white; *n* = 4) and bpV(HOpic) and cisplatin (gray; *n* = 4) treated explants. Percentage of surviving IHC, OHC and undefined hair cells in **(I)** control, **(J)** cisplatin and **(K)** bpV(HOpic) and cisplatin treated cochlear explants. Statistical significance was determined using the Student’s *t*-test and error bars are in SD.

To induce cochlear damage, murine cochlear explants were exposed to cisplatin. Cochleas were obtained from early post-natal day (P) 4–6 mice and cultured for a day to allow for recovery before addition of 10 μM bpV(HOpic). After bpV(HOpic) treatment, cochleae were exposed to 20 μM of cisplatin for 24 h before cisplatin and bpV(HOpic) were removed. Fresh medium without cisplatin or bpV(HOpic) was added to the cochlear cultures. The cochlear explants were allowed to recover for an additional 3 days, fixed and subjected to immunostaining (Figure 3A). Cochleae were immmunostained with antibodies against MYO7A to label IHC and OHC bodies while phalloidin highlighted the actin filled hair bundles and the actin filaments in the sensory epithelium (Figure 3B). In control untreated cochlear explants, the typical one row of IHC and three rows of OHC could be observed. The two types of hair cells are separated by the tunnel of Corti formed by inner and outer pillar cells. The heads of the pillar cells converge on the surface of the sensory epithelium and can be seen as phalloidin labeling between the IHCs and the first row of OHCs (Figure 3B). IHCs and OHCs were defined by their relative location to the heads of the pillar cells. Treatment of cochlear cultures with 20 μM cisplatin for 24 h resulted in the lack of MYO7A labeled hair cell bodies in the sensory epithelium along with the loss of phalloidin marked hair bundles (Figure 3C). Due to the disruption in the microarchitecture of the sensory epithelium, it was sometimes difficult to distinguish between IHCs and OHCs. Pretreatment of cochlear explants with 10 μM bpV(HOpic) displayed increased numbers of surviving MYO7A labeled hair cells with phalloidin marked hair bundles (Figure 3D). In some regions, the IHC and OHC organization could still be discerned.

Since cisplatin-induced toxicity has different effects on IHC and OHCs (Schacht et al., 2012), the percentages of the two different types of hair cells were separately quantified. Surviving IHCs and OHCs were distinguished based on their location relative to where the heads of the pillar cells were located. To determine the effects of IHC and OHC survival after bpV(HOpic) treatment, hair cells were counted along the length of the cochlea. The cochlea was partitioned into three segments, the base, middle and the apex. Surviving hair cells were then represented as a percentage of MYO7A labeled hair cells relative to untreated samples. Treatment of cisplatin showed a significant reduction of MYO7A labeled IHCs along the length of the cochlea relative to controls. The percentage of IHCs after cisplatin damage significantly dropped from 100% to 23.0 ± 5.2% in the base (*p* < 1 × 10^−2^), 32.3 ± 6.6% in the middle (*p* < 1 × 10^−2^) and 40.1 ± 5.7% in the apex (*p* < 1 × 10^−2^). After pretreatment with bpV(HOpic), the percentage of surviving IHCs compared to cisplatin treatment alone significantly increased to 44.8 ± 4.8% in the base (*p* < 0.05), 58.1 ± 6.5% in the middle (*p* < 0.05) and 56.8 ± 5.2% in the apex (*p* < 0.05; Figure 3E). On average, cisplatin treatment significantly decreased IHC levels from 100 ± 9.5% to 32.0 ± 4.4% (*p* < 1 × 10^−3^). Before cisplatin treatment, addition of bpV(HOpic) significantly increased the percentage of IHCs to 53.3 ± 4.2% (*p* < 1 × 10^−2^; Figure 3F). These results suggest that addition of bpV(HOpic) and activation of PI3K signaling increases IHC survival after cisplatin damage.

For OHCs, addition of cisplatin significantly decreased the percentage of MYO7A labeled OHCs from 100% to 3.7 ± 1.0% in the base, 6.7 ± 0.9% in the middle and 5.2 ± 1.3% in the apex. Pretreatment with bpV(HOpic) increased the percentage of surviving OHCs to 11.8 ± 2.8% in the base (*p* < 0.05), 20.2 ± 2.9% in the middle (*p* < 1 × 10^−2^) and 16.1 ± 2.8% in the apex (*p* < 0.05; Figure 3G). On average, cisplatin treatment significantly decreased percentage of OHCs from 100 ± 13.9% to 5.2 ± 1.94% (*p* < 1 × 10^−4^), while the addition of bpV(HOpic) significantly increased the percentage to 16.2 ± 2.7% (*p* < 0.05; Figure 3H). Although bpV(HOpic) promoted OHC survival, OHC loss was still more prominent than IHC loss as previously described (Schacht et al., 2012). Our results demonstrate that activation of PI3K signaling using bpV(HOpic) can increase both IHC and OHC survival after cisplatin-induced damage.

In addition to hair cell loss, we noticed that the microarchitecture of the sensory epithelium was altered and typical hair cell organization was disrupted. Some hair cells could not be unambiguously characterized as IHCs or OHCs. To quantify this effect, surviving IHCs or OHCs were characterized based on their location to the heads of pillar cells. Hair cells that could not be categorized were labeled as undefined. In untreated cells, 25.9 ± 1.5% and 74.1 ± 6.1% of hair cells corresponded to IHCs and OHCs respectively while no undefined hair cells were observed (Figure 3I). These percentages correlate well with the proportion of 1 IHC to 3 OHCs that are normally present in the cochlea. Treatment of cisplatin resulted in 21.8 ± 5.9% IHCs, 31.4 ± 8.5% OHCs with a large population, 46.8 ± 9.3% of undefined hair cells (Figure 3J). Addition of bpV(HOpic) before treatment with cisplatin resulted in 26.7 ± 3.8% IHCs, 64.8 ± 19.6% OHCs and 8.5 ± 2.8% undefined hair cells (Figure 3K). The data suggest that application of bpV(HOpic) to cochlear cultures could activate PI3K signaling in multiple cell types including supporting cells. After activating PI3K signaling, the cochlear cytoarchitecture is maintained in some regions of the sensory epithelium where IHCs can be distinguished from OHCs even after cisplatin damage. We hypothesize that activation of PI3K signaling increases survival of supporting cells by increasing CHK1 activity. Increased supporting cell survival helps provide oto-protection and maintain hair cell organization after cisplatin damage. To directly address whether activation of PI3K signaling increases phosphorylation of CHK1 promotes supporting cells survival after cisplatin damage, a genetic approach was employed.

### Genetic Activation of PI3K Signaling in Supporting Cells

**Figure 4:**
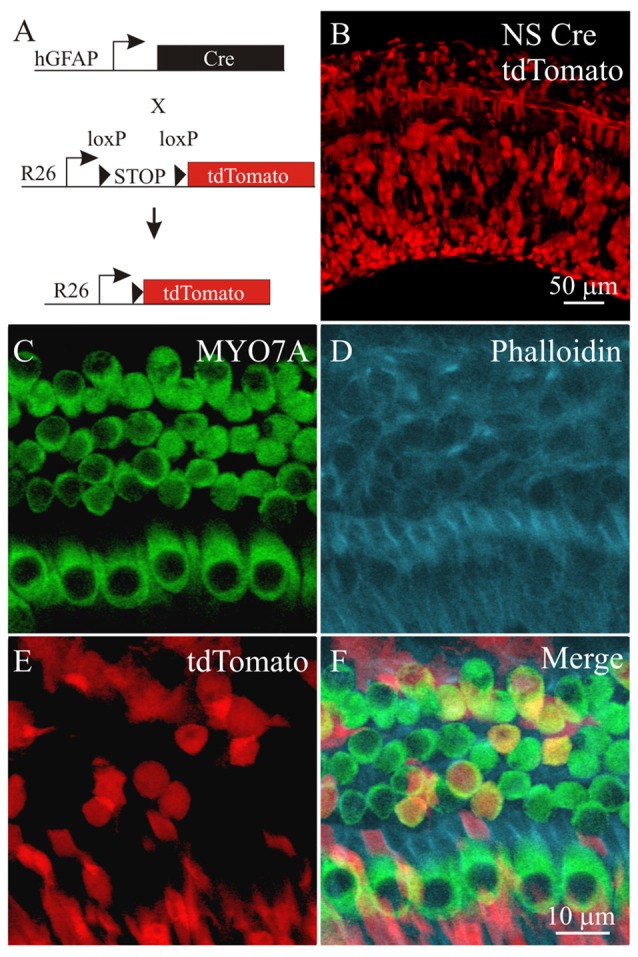
**Labeling cochlear cells using a neural subset (NS) Cre tdTomato reporter mouse. (A)** Diagram of the NS Cre recombinase transgene using a fragment of the human GFAP (hGFAP) promoter driving Cre recombinase and a STOP floxed tdTomato reporter inserted in the ROSA26 (R26) locus. **(B)** tdTomato fluorescence from NS Cre tdTomato cochlea. Confocal section of the cochlear sensory epithelium labeled with **(C)** MYO7A, **(D)** phalloidin, **(E)** tdTomato from the NS Cre tdTomato reporter mouse. **(F)** Merged image (*n* = 4).

To genetically activate PI3K signaling, a *Pten* conditional knock out mouse model was used. PTEN normally dephosphorylates PIP_3_ to PIP_2_ and by ablating the *Pten* gene, PIP_3_ levels increase to activate PI3K signaling (Cantley and Neel, 1999). To target cochlear cell types for Cre mediated excision of *Pten*, we used a neuronal-subset (NS) Cre recombinase mouse that was generated using a promoter fragment of the human *GFAP* promoter ablation to allow cell type specific knockout of *Pten* (Backman et al., 2001; Ljungberg et al., 2009). In these mice, expression of Cre recombinase under the ROSA26 promoter (R26) mediates excision of a loxP flanked STOP cassette and allows expression of the tdTomato red fluorescent protein (Madisen et al., 2010; Figure 4A). To confirm that NS Cre mice could mediate loxP excision in cochlear cell types, a NS Cre tdTomato reporter animal was generated to serve as a control. Fluorescent images of cochleae obtained from NS Cre tdTomato animals showed mosaic expression tdTomato fluorescence in many cochlear cell types (Figure 4B). To determine whether hair cells and supporting cells were labeled with tdTomato, cochlear explants were immmunostained using MYO7A antibodies (Figure 4C) and with phalloidin (Figure 4D). The tdTomato fluorescence (Figure 4E) marked MYO7A labeled hair cells as well as supporting cells that reside adjacent to both IHCs and OHCs (Figure 4F). Analysis of sparsely labeled cells allowed us to determine the direct effect of activating PI3K signaling in supporting cells. These observed effects are cell autonomous and independent of activating PI3K signaling in surrounding cell types.

### Activation of PI3K Signaling Promotes Supporting Cell and Hair Cell Survival

**Figure 5:**
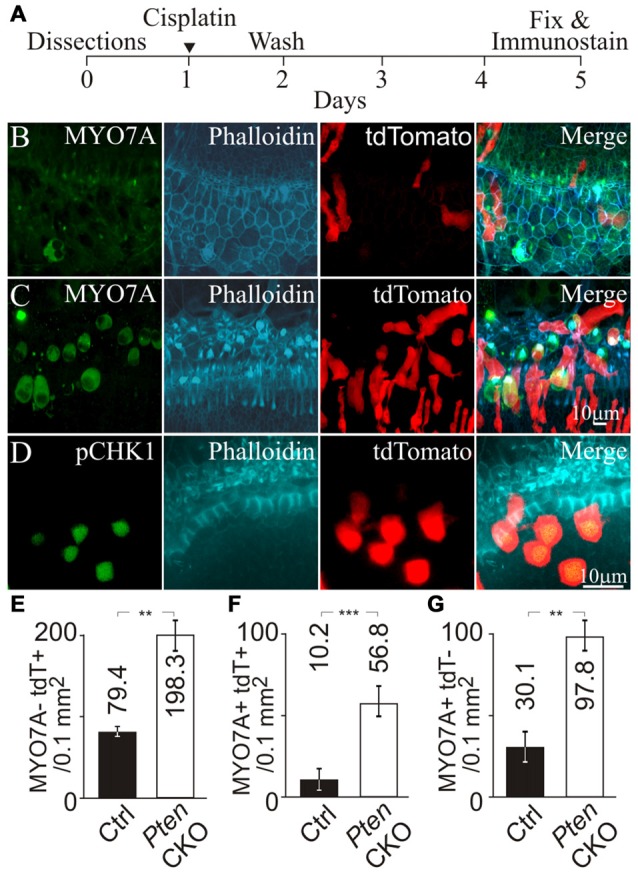
**Hair cell survival after genetic activation of PI3K signaling and cisplatin damage. (A)** Timeline of cochlear explant cultures describing the addition of 10 μM cisplatin treatment for cochlear explants. MYO7A, phalloidin, tdTomato and merged fluorescent images from **(B)** NS Cre tdTomato (control) after cisplatin treatment (*n* = 4) and **(C)** NS Cre *Pten* cKO tdTomato (*Pten* cKO) cochleae after cisplatin treatment (*n* = 4). **(D)** pCHK1, phalloidin, tdTomato and merged fluorescent images from *Pten* cKO mouse after cisplatin damage. **(E)** Density (cells/0.1 mm^2^) of MYO7A- tdTomato+ supporting cells in control (*n* = 4) and *Pten* cKO cochleae (*n* = 4). **(F)** Density of MYO7A+ tdTomato+ hair cells in control (*n* = 4) and *Pten* cKO cochleae (*n* = 4). **(G)** Density of MYO7A+ tdTomato- hair cells in control (*n* = 4) and *Pten* cKO cochleae (*n* = 4). Statistical significance was determined using the Student’s *t*-test and error bars are in SD.

Next, we tested whether activation of PI3K signaling increased CHK1 phosphorylation in supporting cells to promote cell survival after cisplatin damage. The *Pten* conditional knockout allele was introduced into the NS Cre tdTomato animals to generate NS Cre *Pten* cKO tdTomato (*Pten* cKO) animals. These animals allowed activation of PI3K signaling by *Pten* deletion and fluorescently marked cochlear supporting cells with tdTomato expression. To induce ototoxic damage, cochlear explants from NS Cre tdTomato (control) and *Pten* cKO animals were treated with cisplatin. Cisplatin was removed after 24 h and the cochlea explant was replenished with fresh medium without cisplatin. Explants were allowed to recover for an additional 3 days before being fixed and subjected to immunolabeling (Figure 5A). Explants were immunostained for MYO7A and phalloidin to mark the hair cell bodies and the hair bundle. In cisplatin treated control cochlea, few MYO7A labeled hair cells with phalloidin labeled hair bundles and tdTomato marked supporting cells were observed (Figure 5B). The merged image showed disorganized surviving hair cells in the sensory epithelium. In contrast, cisplatin treatment of *Pten* cKO cochlea showed the presence of more MYO7A labeled hair cells with phalloidin labeled hair bundles surrounded by tdTomato expressing supporting cells (Figure 5C). To determine if the increased numbers of tdTomato labeled supporting cells could be due to increased CHK1 phosphorylation after cisplatin damage and activation of PI3K signaling, *Pten* cKO cochleae were immmunostained for pCHK1 after cisplatin damage. Increased nuclear pCHK1 could be observed in tdTomato labeled cells after cisplatin damage (Figure 5D). Increased pCHK1 levels could activate the DNA damage response and improve supporting cell survival.

To ascertain whether increased pCHK1 levels could promote supporting cell survival, we determined the density of surviving supporting cells after cisplatin treatment in control and *Pten* cKO animals. To quantify the presence of supporting cells after cisplatin damage, MYO7A- tdTomato+ cells were counted. A significant increase of supporting cell density from 79.4 ± 2.3 to 198.3 ± 20.1 cells/0.1 mm^2^ (*p* < 1 × 10^−2^) was observed when comparing controls to *Pten* cKO cochleae (Figure 5E). These results demonstrate that *Pten* deletion and subsequent activation of PI3K signaling increases the number of remaining supporting cells after cisplatin damage. As an internal control, MYO7A+ tdTomato+ hair cells were counted to determine whether activated PI3K signaling increases hair cell survival. A significant increase in the density of MYO7A+ tdTomato+ hair cells from 10.2 ± 5.3 to 56.8 ± 8.1 cells/0.1 mm^2^ (*p* < 1 × 10^−3^) was observed when comparing controls to *Pten* cKO animals (Figure 5F). These results are consistent to previous observations that activated PI3K signaling promotes hair cell survival (Jadali and Kwan, 2016). In addition, we also observed many MYO7A+ tdTomato- hair cells. A significant increase in the number of MYO7A+ tdTomato- hair cells from 30.1 ± 8.3 to 97.8 ± 8.1 cells/0.1 mm^2^ (*p* < 1 × 10^−2^) was observed when comparing controls to *Pten* cKO animals (Figure 5G). These results suggest that hair cell survival could not be explained by a simple cell autonomous effect of activated PI3K signaling and increased pCHK1 levels. Since there was an increased density of supporting cells after cisplatin damage, one possibility was that the presence of more supporting cells may indirectly improve hair cell survival after cisplatin damage. To test this hypothesis, we determined whether there was an increased number of supporting cells in the proximity of surviving hair cells.

### Surviving Hair Cells Reside in Close Proximity to Supporting Cells with Activated PI3K Signaling

**Figure 6:**
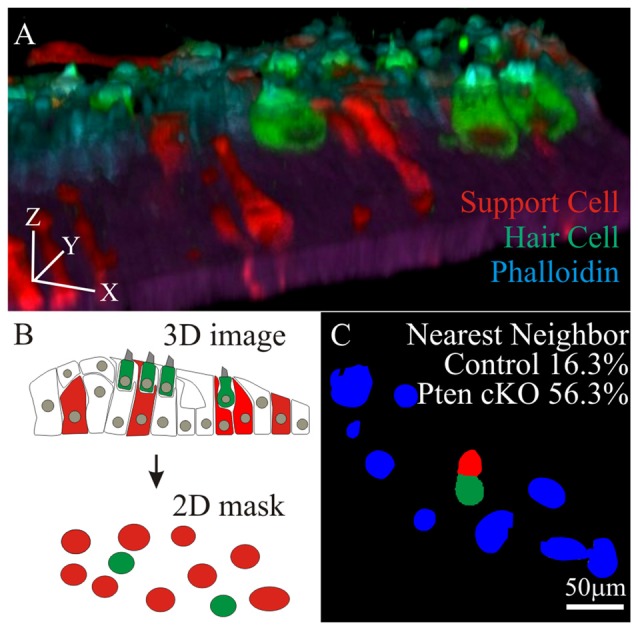
**Nearest neighbor analysis of supporting cell to surviving hair cells. (A)** 3D rendering of a confocal stack with MYO7A and phalloidin labeled hair cells along with tdTomato labeled supporting cell **(B)** 3D images were compressed into a maximal intensity projection to generate a 2D mask for hair cells (green) and tdTomato labeled supporting cells (red). **(C)** Using the 2D masks, percent of surviving hair cells (green) that reside adjacent to supporting cells (red) were determined in control (*n* = 4) and *Pten* cKO cochleae (*n* = 4). All remaining cells in the field of view were labeled blue.

To test if increased hair cell survival correlates to the increased density of neighboring supporting cells, cochlea from control and *Pten* cKO animals were treated with cisplatin and labeled with MYO7A and phalloidin. To reduce the complexity of the analysis, regions along the cochlea lacking tdTomato hair cells were used. In these regions, MYO7A and phalloidin marked surviving hair cells while tdTomato labeled supporting cells. Confocal stacks were used to generate 3D renderings of the region of interest to ensure that only tdTomato supporting cells were present (Figure 6A). Using the 3D confocal image, the image was collapsed to generate 2D masks of hair cells (green) and supporting cells (red; Figure 6B). Using the combined masks, individual surviving hair cells (green) were identified before determining whether a supporting cell (red) resides next to the hair cell. All remaining neighboring cells in the field of view were marked in blue. In controls, 16.3 ± 5.8% of MYO7A labeled hair cells reside adjacent to tdTomato supporting cells. In *Pten* cKO cochleae, 56.3 ± 8.3% of MYO7A labeled hair cells reside next to a PI3K activated tdTomato supporting cells (Figure 6C). A significant increase in the percentage of hair cells adjacent to supporting cells was observed in *Pten* cKO cochleae compared to controls (*p* < 1 × 10^−2^). These data show that many supporting cells with activated PI3K signaling reside in close proximity to surviving hair cells after cisplatin damage.

**Figure 7:**
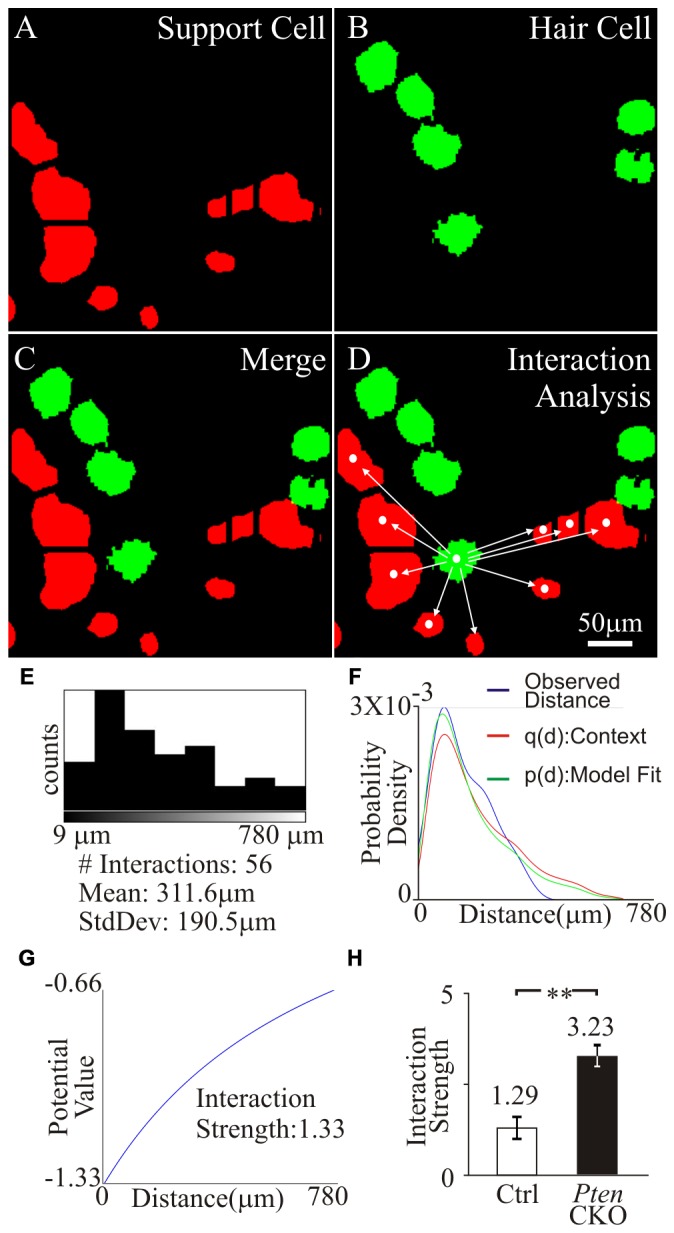
**Interaction strength of supporting cell and surviving hair cells**. Region containing tdTomato+supporting cell and MYO7A+ tdTomato- hair cells were analyzed. **(A)** Supporting cell masks were generated from tdTomato labeled supporting cells (red). **(B)** Hair cell masks were generated from MYO7A labeled hair cells (green). **(C)** Merged image of supporting cell and hair cell masks. **(D)** Centroid of hair cell and supporting cells were generated from masks. Distance between an individual hair cell centroid to all supporting cell centroids in the field of view was determined. **(E)** Number of cell interactions within a field of view and the mean distance between the centroid of a hair cell to multiple supporting cells was displayed in the histogram. **(F)** The observed distances between a hair cell and supporting cells were fitted into probability density, p(d), and an interaction potential model, q(d). **(G)** Interaction strength between hair cell and supporting cells was calculated from the interaction potential q(d). **(H)** Comparison of the interaction strength between surviving hair cells and control supporting cells (*n* = 10) or surviving hair cells and *Pten* cKO supporting cells (*n* = 10) after cisplatin treatment. Statistical significance was determined using the Student’s *t*-test and error bars in SD.

Next, we wanted to determine if the distribution of tdTomato marked supporting cells to surviving MYO7A hair cells in control and *Pten* cKO cochleae were different. To determine the distance between supporting cells and hair cells, an individual hair cell was identified and the distance to all supporting cells in the field of view was measured. To accomplish this, the 2D masks generated from tdTomato expressing supporting cells (Figure 7A) and MYO7A labeled hair cells (Figure 7B) were used to identify the different cell types. The masks were merged into a single image to show the location of hair cells relative to supporting cells (Figure 7C). To determine the spatial distribution between the hair cells and supporting cells, a cellular “interaction analysis” was performed (Helmuth et al., 2010). Centroid determination was done to convert masks that represent individual cell bodies into single points that correspond to the center of the mask. The distance from each centroid corresponding to a hair cell was measured to the centroid of all other supporting cells in the field of view (Figure 7D). The distribution of distances between a single hair cell and supporting cells were measured and plotted as a histogram. The number of interactions and the average distance between hair cell and supporting cells were determined (Figure 7E). The distribution of tdTomato labeled supporting cell relative to surviving hair cells contains information about potential oto-protective effects of supporting cells. The distribution of supporting cells to hair cells would show a stronger “cellular interaction” if supporting cells promoted hair cell survival. The “cellular interactions” between hair cells and supporting cells can be modeled as an interaction potential between the two sets of centroids. Using the centroids obtained from the cell masks, the observed distances between hair cells and supporting cells represented a distribution pattern. From the distribution pattern, a probability density function q(d) of the observed nearest neighbor distances, was calculated to represent the likelihood of a “cellular interaction”. The probability density function was then modeled as an interaction potential p(d), to describe the likelihood that the distribution was due to “cellular interactions” (Figure 7F). The interaction potential p(d) was used to described the “cellular interaction” strength, to determine if objects of a specific distribution had an effect on each other (Figure 7G). A significant increase in interaction strength between tdTomato labeled supporting cell and surviving hair cells was observed in control (1.29 ± 0.4) compared to *Pten* cKO (3.23 ± 0.5) cochleae (*p* < 1 × 10^−2^; Figure 7H). The increased interaction strength in *Pten* cKO cochlea relative to control suggests that the distribution of supporting cells with activated PI3K signaling are highly correlated to the increased numbers of surviving hair cells.

## Discussion

**Figure 8:**
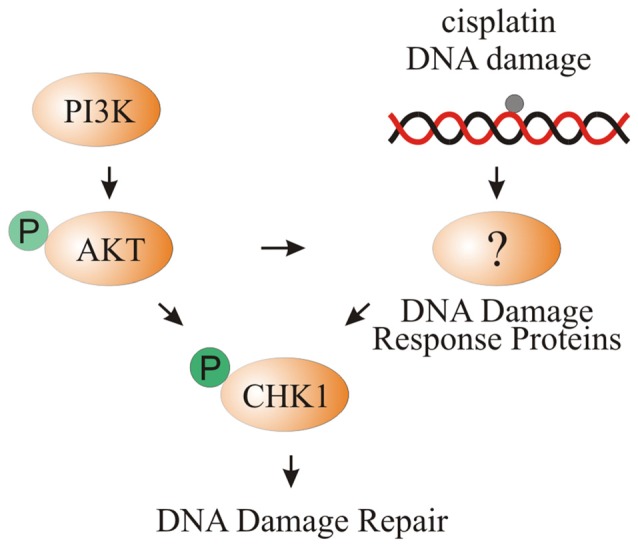
**Model of CHK1 phosphorylation through cisplatin damage and PI3K signaling**. Activation of PI3K signaling either by bpV(HOpic) or *Pten* deletion results in increased AKT phosphorylation. DNA damage caused by cisplatin activates DNA damage response proteins that increase pCHK1 levels. Activated AKT can directly or indirectly increase pCHK1 levels by activating the DNA damage response proteins. Together, PI3K activation and cisplatin-induced DNA damage results in increased pCHK1 to promote supporting cell survival.

In this study, we showed increased phosphorylation of CHK1 after cisplatin damage and activation of PI3K signaling. Activation of CHK1 promotes survival of differentiating iMOP cells. Using cochlear explant cultures, increased phosphorylation of CHK1 after cisplatin damage and activation of PI3K signaling was observed in supporting cells. We propose that increased PI3K signaling activates AKT which could directly phosphorylate CHK1 or indirectly increase pCHK1 levels through DNA damage response proteins such as ATR. Activation of CHK1 allows supporting cells to repair cisplatin-induced DNA damage (Figure 8).

### CHK1 Is Downstream of PI3K/AKT Signaling

Our results suggest that activating the PI3K/AKT signaling pathway increases phosphorylation of CHK1 after cisplatin damage in supporting cells. These results are similar to reports in other systems where CHK1 is phosphorylated by AKT (Jin et al., 2005; Kurosu et al., 2013). Phosphorylation of CHK1 serves as a checkpoint protein that arrests cells at the G2/M phase of the cell cycle after DNA damage (Liu et al., 2000; Takai et al., 2000) and has also been implicated in DNA damage response (Sorensen et al., 2005; Shimada et al., 2008). Since supporting cells are post-mitotic, pCHK1 in supporting cells may contribute to the DNA damage response after cisplatin damage. We propose that pCHK1 can promote survival of cochlear supporting cells. Using differentiating iMOP cells, we showed that cisplatin-induced DNA damage results in phosphorylation of CHK1 at serine 345 using phospho-specific antibodies. Phosphorylation of serine 345 is essential for nuclear localization of CHK1 and response to DNA damage (Niida et al., 2007). Increased PI3K signaling as shown by increased pAKT in iMOP cells contributes to the phosphorylation of CHK1. *In vivo*, activation of PI3K signaling in tdTomato labeled supporting cells increased nuclear pCHK1 levels compared to surrounding cells. We propose that the additive effects of CHK1 phosphorylation may help enhance DNA damage response and increase supporting cell survival after cisplatin damage.

### AKT in Cochlear Supporting Cell Survival

AKT (AKT1) belongs to a family of serine/threonine kinases that act downstream of PI3K and participates in a variety of cellular processes to play a critical role in cell survival (Datta et al., 1999). All three AKT isozymes are expressed in the cochlea (Brand et al., 2015). Mounting evidence shows that the PI3K/AKT signaling pathway plays a role in hair cell survival after exposure to ototoxic drugs. Inhibiting PI3K signaling in cochlear explants while treating with gentamicin showed increased hair cell loss compared to gentamicin exposure alone (Chung et al., 2006). Another study demonstrates that dexamethasone protects HCs against TNFα-initiated apoptosis by activating the PI3K/AKT signaling pathway (Haake et al., 2009). Exposure to simvastatin in cochlear explants activates AKT signaling and protects hair cells from gentamicin toxicity (Brand et al., 2011). These studies suggest that activation of PI3K/AKT signaling promotes hair cell survival. In addition to promoting hair cell survival, activation of PI3K signaling may also promote survival of supporting cells as observed by the increased density of supporting cells in *Pten* cKO cochleae compared to controls after cisplatin treatment. We propose that activation of PI3K/AKT signaling contributes to supporting cell survival by CHK1 phosphorylation.

In cancer cells, several studies have established the involvement of AKT in contributing to cell survival by their acquired cisplatin resistance. These cancers cells include samples from ovarian, uterine, small-cell lung cancer, non-small-cell lung cancer and hepatoblastoma. Studies from these different cell lines and cancer cells suggest that cisplatin-induced DNA damage results in AKT phosphorylation of the pro-apoptotic factor Bcl2-associated agonist of cell death (BAD) at Ser136 to suppress cell death and promote cell survival (Datta et al., 1997; Hayakawa et al., 2000). In ovarian cancer cells, cisplatin-induced DNA damage results in activation of AKT and phosphorylation of X-linked inhibitor of apoptosis (XIAP). AKT phosphorylation of XIAP prevents XIAP ubiquitination and degradation in response to cisplatin to promote cell survival (Dan et al., 2004). In small-cell lung cancer cells, AKT phosphorylates the anti-apoptotic protein survivin to protect cells against cisplatin-induced cell death (Belyanskaya et al., 2005). In all, AKT phosphorylation of key molecules such as BAD, XIAP or survivin promotes cell survival after cisplatin damage. We propose that CHK1 is another downstream target of AKT in both iMOP cells and cochlear supporting cells after cisplatin damage.

### Increased Supporting Cell Survival Maintains Cochlear Cytoarchitecture and Indirectly Promotes Hair Cell Survival

We noticed that after cisplatin damage, many hair cells could not be defined as either IHCs or OHCs due to the altered microarchitecture of the sensory epithelium. We propose that disorganization of hair cells may be due to supporting cell loss. Increased survival of supporting cells after activating PI3K signaling helps maintains hair cell organization in the sensory epithelium. In addition to changes in the cytoarchitecture of the sensory epithelium, supporting cell loss may indirectly contribute to hair cell loss. Our study showed increased density of PI3K activated supporting cells that reside in the vicinity of surviving hair cells compared to controls. The increased presence of supporting cells may provide additional oto-protection to hair cells. One mechanism for oto-protection is the release of HSP70 by supporting cells onto nearby hair cells (May et al., 2013). Another potential mechanism of oto-protection is cell-cell signlaing between hair cells and supporting cells. The ERBB signaling pathway is an example that cell types in the cochlea are constantly communicating with each other through cell-cell signaling. ERBB signaling has been observed between SGNs and supporting cells and presence of this signaling pathway is essential for survival of SGNs (Stankovic et al., 2004). Cell-cell signaling between supporting cells and hair cells may be another cellular mechanism that normally maintains hair cell viability. Ablation of inner border cells and inner phalangeal cells that flank IHCs causes loss of IHC and significantly impairs hearing in adult mice (Mellado Lagarde et al., 2014). The study suggests that cellular contact between supporting cells and IHCs may be essential for maintaining hair cell viability. We propose that after cisplatin damage, activation of PI3K signaling increases survival of supporting cells. Through several potential mechanisms including secretion of extracellular molecules or cell-cell signaling, the increased number of supporting cells promotes survival of nearby hair cells. The study implicates supporting cells as a cellular target for preventing hair cell loss.

## Author Contributions

AJ and KYK designed, performed the experiments and wrote the manuscript. Y-LMY helped with cell counts.

## Funding

The work was supported in part by the Duncan and Nancy MacMillan Faculty Development Chair Endowment Fund (KYK) and National Institute on Deafness and Other Communication Disorders (NIH) R01 DC15000 (KYK).

## Conflict of Interest Statement

The authors declare that the research was conducted in the absence of any commercial or financial relationships that could be construed as a potential conflict of interest.
